# The role of the calcofluor white staining in the diagnosis of Acanthamoeba keratitis

**DOI:** 10.1186/s12348-023-00345-2

**Published:** 2023-05-02

**Authors:** Carolin Elhardt, Romina Schweikert, Lennart Maximilian Hartmann, Efstathios Vounotrypidis, Adnan Kilani, Armin Wolf, Christian Maximilian Wertheimer

**Affiliations:** Department of Ophthalmology, University Hospital Ulm, Prittwitzstrasse 43, Ulm, 89075 Germany

**Keywords:** *Acanthamoeba* keratitis, Calcofluor white, Polymerase chain reaction, Microbiological testing

## Abstract

**Purpose:**

*Acanthamoeba* keratitis is often misdiagnosed at disease onset. This study presents data to confirm the diagnosis using calcofluor white (CFW) staining.

**Methods:**

Forty three patients were retrospectively included who presented to the Department of Ophthalmology at the University Hospital Ulm with keratitis between 2000 and 2022. Condition positive cases were diagnosed based on the typical clinical presentation of *Acanthamoeba* keratitis with a positive polymerase chain reaction (PCR). Condition negative were patients with ulcers due to other causing pathogens with a negative *Acanthamoeba* PCR result. The condition was compared with the CFW test results.

**Results:**

After symptom onset, time until presentation was 17 ± 12 days and until diagnosis 27 ± 13 days in the 15 condition positive patients. Among the 35 patients with additional CFW test, 7 patients were condition positive and 28 negative. 5 of the 7 patients were true positive, 2 were false negative. In the 28 condition negative patients, 1 was false positive. Sensitivity of CFW was 71% and specificity 96%. The positive PCR results were available 3.4 ± 2.3 days after corneal scraping, the positive CFW test results on the same day in each case.

**Conclusion:**

Our data demonstrate that diagnosis of *Acanthamoeba* keratitis remains difficult and therapy is initiated late. A positive CFW test confirms the diagnosis as there are almost no false positive results and it was available faster than PCR. In case of a negative CFW test, *Acanthamoeba* keratitis cannot be ruled out because of a high false negative rate.

## Background

*Acanthamoeba* keratitis (AK) is a potentially vision threatening [1, 2] but rare disease [3]. It is caused by *Acanthamoeba*, which are ubiquitous, free-living protozoa. One of the biggest risk factors is the use of contact lenses [4]. It is difficult to diagnose, as AK is often mistaken for other much more common corneal diseases [5] – in a case study, 77% of the patients were first diagnosed with herpes keratitis [6].

AK is difficult to treat, and current treatment is toxic and must be used over a long period of time. Therefore, accurate diagnosis is essential. There are several methods to verify the diagnosis. Different material can be used to test for *Acanthamoeba*, such as corneal swabs or corneal biopsies. One option is to confirm *Acanthamoeba* by polymerase chain reaction (PCR), detecting its cellular and mitochondrial DNA, and to compare the result with clinical findings and possible resistance to other treatments [1].

In addition, cysts and trophozoites can be verified with a variety of staining methods including hematoxylin and eosin (H&E), periodic acid–Schiff (PAS), Gömöri methanamine silver, Giemsa, Gram and acridine orange [7, 8]. However the cysts are reported to be easily mistaken for other cells in several protocols [9]. The calcofluor white (CFW) stain was reported to be a simple and rapid method to detect *Acanthamoeba* [10]. Its chemofluorescent dye has an affinity for polysaccharide polymers in the cyst wall like cellulose and chitin [10]. After staining, it shows a clearly delineated double-walled cyst with apple-green fluorescence and allows differentiation from single membrane corneal cells [10].

There are a few studies in the literature on staining of corneal specimen for *Acanthamoeba* using different staining methods [9], but to the best of our knowledge, there is a lack of determination of the diagnostic value of the CFW test compared to other diagnostic standard methods. Therefore, this study attempts to mathematically calculate sensitivity and specificity to describe the accuracy of a test that indicates the presence or absence of *Acanthamoeba*.

## Methods

### Patient selection and study type

This is a retrospective study of 15 eyes of 15 patients with corneal infection due to *Acanthamoeba* and 28 patients with corneal ulcer due to other infectious reasons, who were used as a negative test group. Data collection was performed in the Department of Ophthalmology at the University Hospital Ulm between 2000 and 2022. The study was approved by the local ethics committee of the University Ulm (Approval ID: 178/21) and adhered to the Declaration of Helsinki.

### Diagnostic and inclusion criterion

Because the diagnosis of AK is difficult, the true condition can never be known with 100% certainty. Therefore, true condition positive cases were diagnosed by using the typical clinical presentation in conjunction with a positive PCR. Condition negative were patients, who suffered from keratitis with a PCR negative result for *Acanthamoeba* and in which other pathogens were identified as a cause for their keratitis. True positive was assumed when the CFW test correctly indicated the presence of *Acanthamoeba*, true negative correctly indicated the absence of *Acanthamoeba* in the CFW test. A test result which wrongly indicated the presence of AK if there was none was false positive. A test result which wrongly indicated absence was false negative.

### Microbiological testing

The CFW test and PCR were performed with corneal scraping. The PCR (LightCycler® 2.0 Instrument, Roche Life Science, Bavaria, Germany) used is specific for pathogens of the *Acanthamoeba* genus and has a sensitivity of about 10 *Acanthamoeba* cysts per milliliter. PCR was conducted as advised by the manufacturer. For the CFW staining, the corneal scrape was placed on a microscope slide (Epredia, Braunschweig, Germany) and air dried. Consecutively, slides were fixed in methanol for 3–5 min and again allowed to dry. Then, 1–3 drops of fungi-fluor solution (Fungi-Fluor Pneumocystis Kit, Polysciences Europe GmbH, Germany) were applied to the slide for 1 min. The slides were rinsed with distilled water. Afterwards, they were placed obliquely in a dark chamber and were air dried. Images were acquired using a fluorescence microscope equipped with a camera (DM4000B, Leica, Wetzlar, Germany) with an excitation at 400–500 nm and a barrier filter at 510–530 nm.

### Clinical follow-up

All patients received intensive topical treatment adapted to the patients’ needs. Regular follow-up examinations at adequate intervals were performed in the outpatient clinic. In case of worsening or perforation, patients were readmitted to the inpatient ward. Baseline characteristics, symptoms, time course, and clinical presentation were also noted.

### Statistical analysis

For data collection and statistical analysis Microsoft Excel 365 (Microsoft, Redmond, Washington) was used.

## Results

### Baseline characteristics, clinical presentation, and follow-up

The mean age of the patients with AK was 32 ± 14 years (13–54 years). 9 out of 15 patients (60%) were female. All 15 patients (100%) were contact lens wearers. The mean follow-up was 247 days. Symptoms had been present in patients for 17 ± 12 days (2–44 days) until first consultation at our clinic. None of the patients had been pretreated with therapy against *Acanthamoeba* at the time of presentation. Various medications had been taken prior to the day of the initial visit, including topical steroids. As soon as the diagnosis *Acanthamoeba* was made, the topical therapy was adjusted and all patients received propamidine isethionate, antibiotic eyedrops and polyhexamethylenbiguanide. Four patients required surgical therapies in addition to topical *Acanthamoeba* therapy. One patient received a penetrating keratoplasty, another one a phototherapeutic keratectomy with following penetrating keratoplasty. A third patient had three penetrating keratoplasties, one with a following amniotic membrane transplantation. In another patient a photo activated chromophore for keratitis crosslinking was performed in addition to topical treatment.

### Microbiological results

**Fig. 1:**
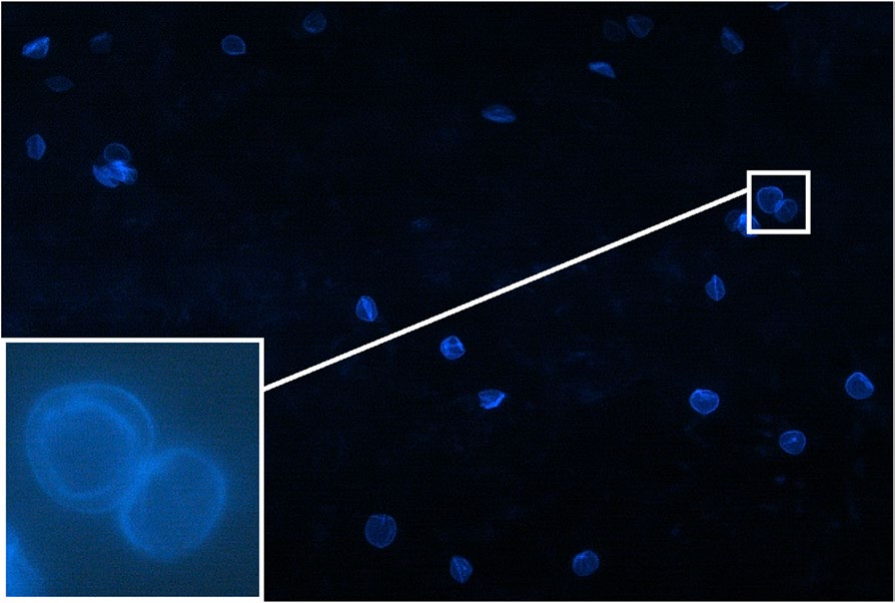
Small, double-walled *Acanthamoeba* structures were detected in the Calcofluor white test from material obtained by corneal scraping

After symptom onset, the diagnosis was available on average after 27 ± 13 days. In comparison to the positive PCR results, which were available 3.4 ± 2.3 days after the performed eye swab, the positive CFW test results were available on the same day in each case. In 5 of the 7 patients (71%) who underwent a CFW staining of the corneal scraping the result was positive: Small, double-walled *Acanthamoeba* structures were detected (Fig. 1).

### Sensitivity and specificity of the calcofluor white test

Five out of the 7 patients that were tested condition positive with a positive PCR combined with a typical clinical presentation also had a positive CFW test in the corneal scraping; 2 were negative in the CFW test. One additional PCR positive cornea was explanted in the course of treatment and the corneal button was tested with H&E, PAS und Giemsa staining, which were negative for *Acanthamoeba*. In addition, this study also looked at patients that were condition negative with a negative PCR test while a CFW test was performed simultaneously. Twenty-eight patients met these criteria. Except in one patient, the CFW staining was also negative in all these condition negative patients. Therefore, the calculated sensitivity of the CFW test performed on corneal scrapings was 71% and the specificity was 96% (Table 1).

**Table 1:** The four outcomes can be formulated in a 2 × 2 contingency table; sensitivity was calculated as 71% and specificity as 96%

Total population: 35		CFW result		Total	Accuracy
		Positive	Negative		
Actual condition	Positive	True positive: 5	False negative: 2	= 7	Sensitivity: 71%
	Negative	False positive: 1	True negative: 27	= 28	Specificity: 96%

## Conclusion

Diagnosis and management of AK can be extremely challenging. *Acanthamoeba* should be considered as a possible cause of keratitis, especially in cases which are resistant to initial treatment. The diagnosis should be confirmed as it is difficult to treat, and current treatment is toxic with a combination of topical diamidines, biguanides and antibiotic eye drops significantly improving the outcome of AK [11]. For pathogen detection, CFW staining or PCR are available among other possible procedures. Besides the above mentioned possible staining methods, confocal microscopy has been shown to have a high sensitivity and specificity for the detection of *Acanthamoeba* [12] and culture is described as an important element for diagnosis of *Acanthamoeba* [13]. This study calculated sensitivity and specificity to describe the accuracy of CFW staining which reports the presence or absence of a *Acanthamoeba*.

In literature, few studies have used CFW staining to detect AK [14] showing clearly delineated double-wall cysts with apple-green fluorescence [10]. Even though evidence is low, it has been described as a reliable and rapid technique for some organisms including *Acanthamoeba* [10]. For this purpose it has also been compared to other staining methods revealing it to be able to detect *Acanthamoeba* even when the other methods failed. For example, in a prospective study, four out of four culture-proven AK were detected by CFW staining, whereas Gram and Giemsa stains were positive in only one of these cases [9]. Another case described the detection of *Acanthamoeba* cysts with retrospective CFW staining after Gram's and Giemsa stains presented only poorly stained trophozoites [15]. In our study, the sensitivity was lower at 71% and we must point out that in case of a negative CFW test, AK cannot be ruled out due to a high false negative rate in our study population. Nonetheless, a positive CFW test is still helpful because the result is available faster than with PCR so the therapy can be initiated more quickly.

We found a high specificity of 96% with only one false positive test result in the control group of keratitis not caused by *Acanthamoeba*. Different results were described by another study, which compared different staining methods (H&E, PAS, Gömöri methanamine silver, Giemsa, Gram, CFW, acridine orange) according to both their staining quality and techniques in nine patients with varying causes of corneal ulcers in culture-proven *Acanthamoeba*, fungal and herpes simplex keratitis. The H&E stain was used as reference for the right diagnosis. The CFW test was only positive in two of the three positive *Acanthamoeba* cases and was the only test with false-positive diagnosis because of simultaneous staining of fungal wall, debris, and basement membrane. Unspecific staining of cotton fibers and dust particles was also observed. Because *Acanthamoeba* and herpes was reported to coexist in keratitis [16], the authors indicate that the stained debris in the herpes case marked as false positive could also be degenerated *Acanthamoeba*. They also pointed out that the size and shape of yeast and cross-sections of hyphae can resemble that of *Acanthamoeba* [7].

PCR, on the other hand, was able to detect *Acanthamoeba* in our study, even when the CFW test was negative. In literature, PCR is reported to have a sensitivity and specificity of up to 100%. In a study, two real-time PCR tests for *Acanthamoeba* detected all 7 culture positive cases and had no false positive results in 37 culture negative cases, presenting a sensitivity and specificity of 100% in this study [2]. One study even reported the PCR test to be more sensitive than culture, presenting 84% (16 of 19 cases) of epithelial biopsies of firm clinically diagnosed AK as PCR positive and only 53% (10 of 19 cases) culture positive while all culture positive cases were also PCR positive. None of the 15 control biopsies were PCR positive [17]. Another study, on the other hand, reported only 24 PCR positive cases of 31 patients (77%) with evidence of *Acanthamoeba* by confocal microscopy [18]. Compared to culture proven and culture negative AK, PCR was also described with a sensitivity of 75% (3 out of 4 eyes) and specificity of 70% (14 out of 20 eyes) as well as with a sensitivity of 71% (10 out of 14 eyes) and specificity of 100% (12 out of 12 eyes) compared to a “definite AK” diagnosis defined as a positive test result on in vivo confocal microscopy, PCR and/or culture as well as disease resolution with therapy against *Acanthamoeba* [12]. Due to the overall high detection rate of PCR, other authors have used real-time PCR as comparison for the testing of other methods such as next-generation sequencing based ribosomal gene detection [19]. However, the results of the PCR require a longer time. To benefit from both a rapid and a potentially more sensitive evaluation, a compromising diagnostic proposal would be to order both CFW staining and PCR in case of a suspected diagnosis of *Acanthamoeba*.

Regarding the clinical course, as mentioned above, AK is often mistaken for other corneal diseases [5] especially because indicative findings like ring infiltrates or perineural infiltrates are not always present [20]. This was also the case in our study, and the pretreatment therapy prior to the first visit at our clinic gives an indication of the initially suspected diagnoses which include bacterial and herpes simplex keratitis. In addition, in literature, about 20–25% of patients with AK need a corneal transplantation [21, 22]. The number of patients in our study requiring a penetrating keratoplasty was 21%, which is within the range reported in literature. The failure of treatment in the patient with multiple penetrating keratoplasties in our study shows once again, that corneal transplantations in patients with *Acanthamoeba* are procedures with significant postoperative complications [22] and are often no definite therapy also due to persistent or reactivation of the infection [23].

Limitations of our study include the limited number of patients due to the rarity of the disease and the short follow up time in some cases. It should be noted that due to the small number of cases, our study can only provide indications. A larger study is necessary to perform further analyses to support our statements.

To conclude, an accurate and rapid microbiological evaluation should be performed if *Acanthamoeba* are suspected. For this purpose, the CFW test offers the possibility of a very rapid test result with the potential for a faster start of therapy since the results are available several days before the PCR. If the CFW test is positive, it confirms the diagnosis, as there are almost no false positive results in our cohort. The result of the CFW staining was available faster than the result of the PCR. In case of a negative CFW test, AK cannot be ruled out due to a high false negative rate.

## Data Availability

The datasets used and/or analysed during the current study are available from the corresponding author on reasonable request.

## Abbreviations

PCR: Polymerase chain reaction
CFW: Calcofluor white
AK: *Acanthamoeba* Keratitis
